# The efficacy of immune checkpoint inhibitors is limited in elderly NSCLC: a retrospective efficacy study and meta-analysis

**DOI:** 10.18632/aging.205328

**Published:** 2023-12-19

**Authors:** Jiaxin Yin, Yuxiao Song, Yang Fu, Wang Jun, Jiazhuo Tang, Zhimin Zhang, Qibin Song, Bicheng Zhang

**Affiliations:** 1 Cancer Center, Renmin Hospital of Wuhan University, Wuhan, China; 2 Department of Oncology, Xiangyang Hospital, Hubei University of Chinese Medicine, Xiangyang, China; 3 Department of Oncology, The First Affiliated Hospital of Shandong First Medical University, Jinan, China

**Keywords:** immune checkpoint inhibitors, non-small cell lung cancer, age, elderly, meta-analysis

## Abstract

Immune checkpoint inhibitors (ICIs) have improved the long-term survival of NSCLC patients. However, the efficacy of ICIs in elderly NSCLC patients remains controversial. We conducted a retrospective study and meta-analysis exploring the efficacy of ICIs in those patients using public databases and RCTs. NSCLC patients were identified into elderly and non-elderly groups by age 75 years. The retrospective study showed significant differences in OS and PFS between non-elderly and elderly patients treated with ICIs (P= 0.029 and 0.027), with reduced efficacy in elderly NSCLC patients. ECOG PS also negatively affected OS in elderly NSCLC patients (P= 0.007). In meta-analysis, the HR for OS in the non-elderly and elderly groups were 0.74 and 0.90, respectively, and the difference between the two age groups was statistically significant (P= 0.025). ICIs resulted in a lower incidence of all-grade (OR= 0.47) and high-grade TRAEs (OR= 0.38) than chemotherapy. Our findings revealed that the survival benefit of ICIs in elderly patients (≥ 75 years) may be lower than in non-elderly patients. In addition, the incidence of TRAEs induced by ICIs was lower than chemotherapy.

## INTRODUCTION

Lung cancer is a significant public health problem and the leading cause of mortality worldwide, with non-small-cell lung cancer (NSCLC) accounting for over 80% [1]. The incidence of NSCLC is more prevalent among the geriatric population, with a median age of 71 years at the newly diagnosed and up to 40% patients aged ≥ 75 years [2]. The Chinese expert consensus defines elderly cancer patients as ≥ 75 years old [3]. Therefore, a critical age of 75 is considered reasonable to differentiate the elderly and non-elderly in NSCLC patients. However, NSCLC patients aged 75 years or older are underrepresented; the percentage of those patients enrolled in clinical trials is extremely low. Many phase II/III clinical trials exclude them, resulting in a lack of evidence for optimal treatment options for such population [4]. Thus, the therapeutic effect in elderly NSCLC patients aged ≥ 75 years deserves more attention.

The notable impact of emerging immune checkpoint inhibitors (ICIs) on the overall survival (OS) and treatment patterns of NSCLC patients is undeniable, which has transformed the conventional treatment regimen for NSCLC patients of all ages, including the elderly. After the approval of ICIs for NSCLC patients, the administration of ICIs increased from 4.7% in 2015 to 45.6% in 2019 [5]. The initial age of NSCLC patients using nivolumab or pembrolizumab was 69, with 27% aged 75 years and older [6]. However, there is limited information from prospective trials for the efficacy of ICIs in NSCLC patients aged ≥ 75 years, and most of evidence comes from subgroup analyses. It has been observed in clinical practice that NSCLC patients of different ages respond differently to ICIs, and elderly patients tend to have poor outcomes. In addition, conclusions from existing studies are not entirely consistent regarding ICIs in elderly patients.

Elderly patients with NSCLC are confronting unique challenges due to age-related changes in their bodies, such as cellular senescence, epigenetic alterations, and loss of proteostasis (also known as immunosenescence) [7]. Aging of the innate and adaptive immune systems secondary to immunosenescence may reduce the efficacy of ICIs [8]. Additionally, chronic diseases and consequent organ dysfunction often co-exist in elderly patients, which may lead to poor treatment tolerance and an increase in treatment-related adverse events (TRAEs). Thus, the treatment goals for them should not only consider cancer control, but also the quality of life. However, few studies focus on the administration of ICIs to elderly NSCLC patients. Taking nearly half of NSCLC patients who are 75 years and older in the real world into account [9, 10], there is an urgent need for high-quality studies exploring the potential survival benefits of ICIs in those patients.

Given the above, this study aims to summarize the available data on the efficacy and safety of ICIs in aged ≥ 75 years old NSCLC patients from clinical trials. We used clinical information of NSCLC patients in the cBioPortal for Cancer Genomics database (http://www.cbioportal.org/) [11] to reflect the efficacy of ICIs in real-world elderly patients. However, the sample size was not convincing enough, so we performed a meta-analysis of randomized controlled trials (RCTs) to assess the long-term survival of ICIs in those patients.

## RESULTS

### Retrospective efficacy study

A total of 336 NSCLC patients treated with ICIs were identified in this study. The raw data from the cBio website are summarized in Supplementary Appendix 1. The demographic and clinical characteristics of these patients are summarized in Table 1. A total of 60 (17.9%) NSCLC patients aged 75 years or older were enrolled in this study. Most patients had a tumor histological type of lung adenocarcinoma, an Eastern Cooperative Oncology Group (ECOG) performance status (PS) of 1, and were mostly treated with anti-PD-1 monotherapy.

**Table 1:** Characteristics and univariate analysis of mOS in NSCLC patients treated with ICIs from the cBio database.

**Clinical characteristic**	**Patient and percentage (%)**	**mOS (months)**	**P-value**
Total case	133 (100%)	4.6	-
Age			
Aged < 75 years old	106 (79.7%)	5.2	0.029^*^
Aged ≥ 75 years old	27 (20.3%)	3.6	
Sex			
Male	60 (45.1%)	4.0	0.955
Female	73 (54.9%)	4.7	
Tumor Histology			
Adenocarcinoma	114 (85.7%)	4.3	0.624
Squamous	19 (14.3%)	5.9	
ECOG PS			
ECOG 0 ECOG 1 ECOG 2 ECOG 3	12 (9.0%) 104 (78.2%) 17 (12.8%) 0 (0%)	8.9 4.6 1.9	0.007^**^
Types of ICIs			
Anti-PD-1 Anti-PD-L1 Anti-PD-1/L1 + Anti-CTLA-4	101 (75.9%) 29 (21.8%) 3 (2.3%)	4.6 5.0 2.4	0.665

*P < 0.05, **P < 0.01.

**Figure 1:**
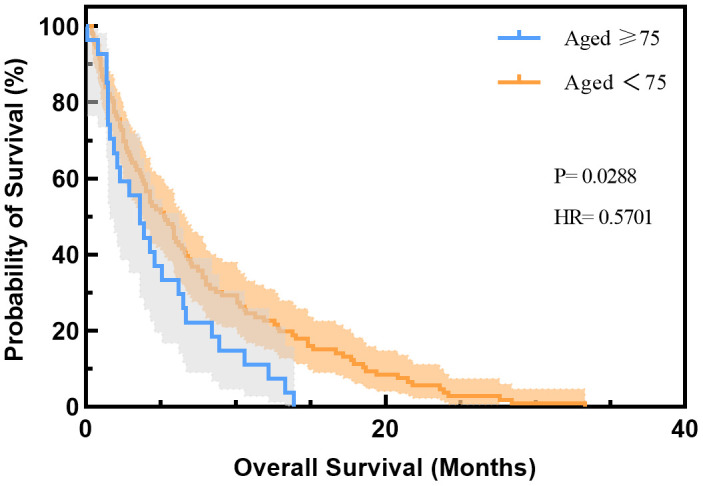
**Overall survival of NSCLC patients treated with ICIs based on age group in the cBio database.** HR, hazard ratio.

OS data were available for 133 of 336 NSCLC patients, 27 of whom were aged ≥ 75 years. The results of the univariate analysis of median OS (mOS) based on clinical characteristics are described in Table 1. The mOS for the whole NSCLC patients treated with ICIs was 4.6 months (range 3.2 months to 6.0 months). NSCLC patients aged < 75 years had a longer OS of 5.2 months (range 3.7 months to 6.7 months), while those aged ≥ 75 years had a shorter OS of 3.6 months (range 1.9 months to 5.3 months). The univariate analysis showed that age significantly affects OS in NSCLC patients treated with ICIs (P= 0.0288) (Figure 1). In addition, ECOG PS significantly affected OS with 8.9 months, 4.6 months, and 1.9 months for patients with PS= 0, 1, and 2, respectively (P= 0.0074) (Supplementary Figure 1). No difference in OS was observed in subgroups defined by sex, tumor histology, and type of ICIs. Multivariate analysis revealed that ECOG PS was an independent risk factor for OS, with p-values of 0.032 and 0.005 for NSCLC patients with PS of 1 and 2, respectively, and hazard ratio (HR) of 1.95 (95% CI: 1.06 to 3.58) and 2.99 (95% CI: 1.40 to 6.4), respectively. Although age was insignificant (P= 0.068), the HR was 1.51 (95% CI: 0.97 to 2.35), indicating that elderly NSCLC patients using ICIs have a 1.5-fold higher risk of death than non-elderly patients.

Progression-free survival (PFS) data were available for 296 of 336 NSCLC patients, 51 of whom were aged ≥ 75 years. The results of the univariate analysis of median PFS (mPFS) based on clinical characteristics are displayed in Supplementary Table 2. The mPFS of patients aged < 75 years was 3.2 months, while mPFS of patients aged ≥ 75 years were only 2.3 months. Univariate analysis showed that age significantly affected PFS in NSCLC patients (P= 0.027) (Supplementary Figure 2). While there was no statistical difference in the effect of sex and tumor histology on PFS. A statistically significant difference in ECOG and types of ICIs was validated (P < 0.001 and 0.020, respectively) (Supplementary Table 2).

### Meta-analysis

### Search results and patient characteristics

**Figure 2:**
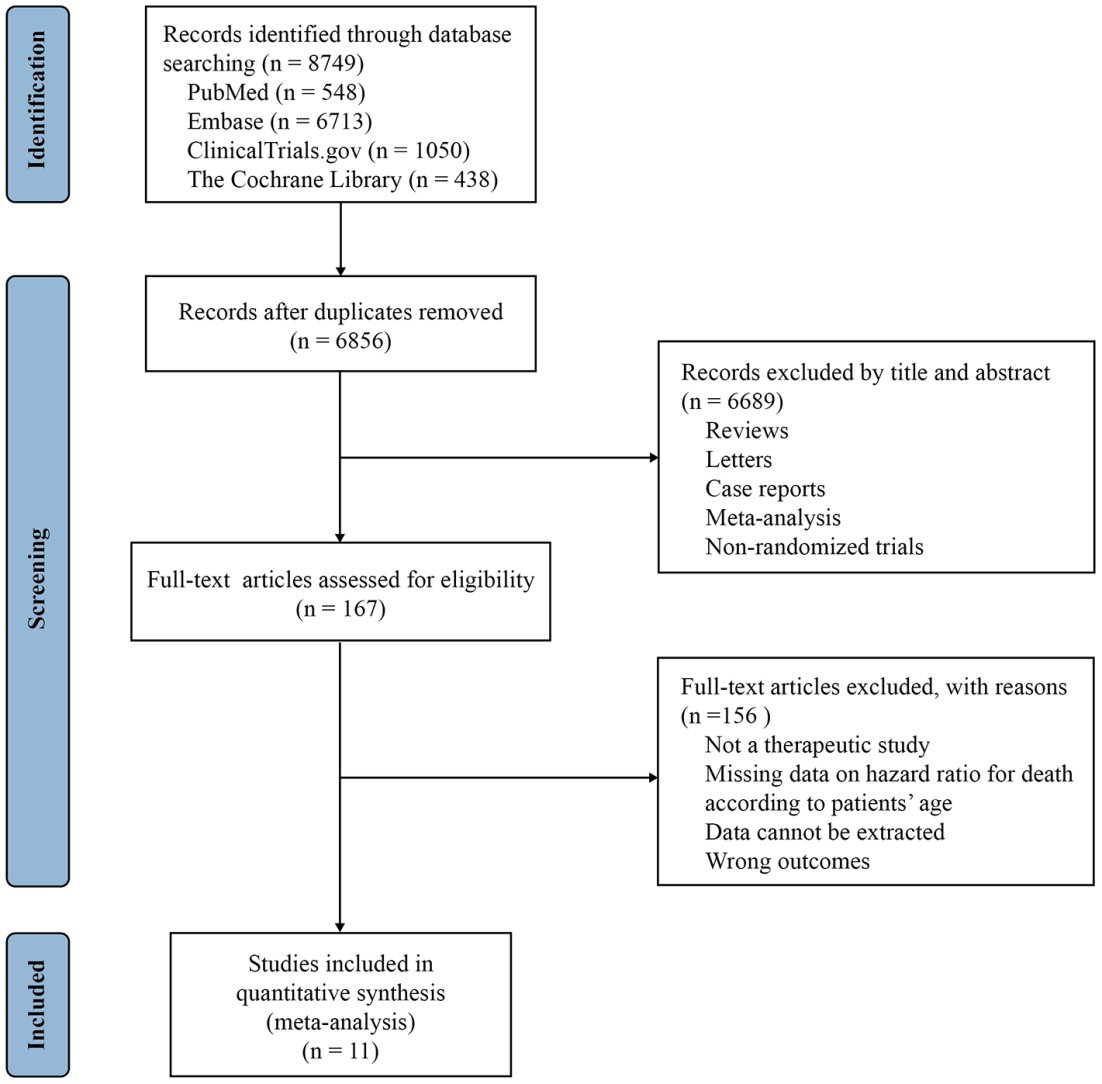
Study selection PRISMA flow diagram.

A total of 8749 publications were initially aligned with our fundamental search strategy. Following a meticulous screening process (Figure 2), 1 phase II/III and 10 phase III RCTs of NSCLC eligible for meta-analysis emerged, among which three were for pembrolizumab, two for nivolumab, two for the combination of nivolumab and ipilimumab, two for atezolizumab, one for ipilimumab and one for avelumab [12–19]. HR values corresponding to the 10 mg/kg dose of pembrolizumab were extracted from the KEYNOTE-010 study [20]. Eight trials investigated anti-PD-1/L1, one investigated anti-CTLA-4, and two investigated anti-PD-1 plus anti-CTLA-4 therapy in experimental arms. A total of 7459 patients were incorporated into the meta-analysis, including 761 patients (10.2%) aged 75 years or older. The detailed baseline characteristics of each trial are shown in Table 2.

**Table 2:** Baseline characteristics of 11 randomized controlled studies.

**Studies**	**Study number**	**Phase**	**Treatment lines**	**Experimental arm**	**Control arm**	**Patients number**	**Median age (years)**	**N < 75 years**	**N** ≥ **75 years**	**Median follow-up (months)**
Paz-Ares, 2021	CheckMate 9LA (NCT03215706)	III	1	Nivolumab plus ipilimumab with chemotherapy	Chemotherapy	719	65 (59 to 70)	649	70	9.7
Borghaei, 2015	CheckMate 057(NCT01673867)	III	2	Nivolumab	Docetaxel	582	62 (21 to 85)	539	43	13.2
Hellmann, 2019	CheckMate 227(NCT02477826)	III	1	Nivolumab plus ipilimumab	Chemotherapy	793	64 (26 to 87)	712	81	29.3
Barlesi, 2018	JAVELIN Lung 200 (NCT02395172)	III	2	Avelumab with antihistamine and paracetamol	Docetaxel	529	64 (58 to 89)	479	50	18.3
Socinski, 2021	IMpower150 (NCT02366143)	III	1	Atezolizumab with carboplatin and paclitaxel	Chemotherapy	697	63 (31 to 89)	627	65	32.4
Govindan, 2017	Study 104(NCT01285609)	III	2	Ipilimumab with carboplatin and paclitaxel	Chemotherapy	749	64 (28 to 84)	678	71	12.5
Rittmeyer, 2017	OAK(NCT02008227)	III	2	Atezolizumab	Docetaxel	850	64 (33 to 85)	762	88	21
Brahmer, 2015	CheckMate 017(NCT01642004)	III	2	Nivolumab	Docetaxel	272	63 (39 to 85)	243	29	11
Herbst, 2015	KEYNOTE 010(NCT01905657)	III	2	Pembrolizumab	Docetaxel	689	63 (56 to 89)	943	90	13.1
Reck, 2016	KEYNOTE 024(NCT02142738)	III	1	Pembrolizumab	Chemotherapy	305	65 (33 to 90)	260	45	11.2
De-Castro, 2022	KEYNOTE 042(NCT02220894)	III	1	Pembrolizumab	Chemotherapy	1274	63 (25 to 89)	1145	129	61.1

### Risk of bias

The mean Jadad score was 3.4, with a range of 3 to 5. Detailed information pertaining to randomization, blinding, and accounting is presented in Supplementary Table 3. All trials met high quality.

### Primary outcome: overall survival

**Figure 3:**
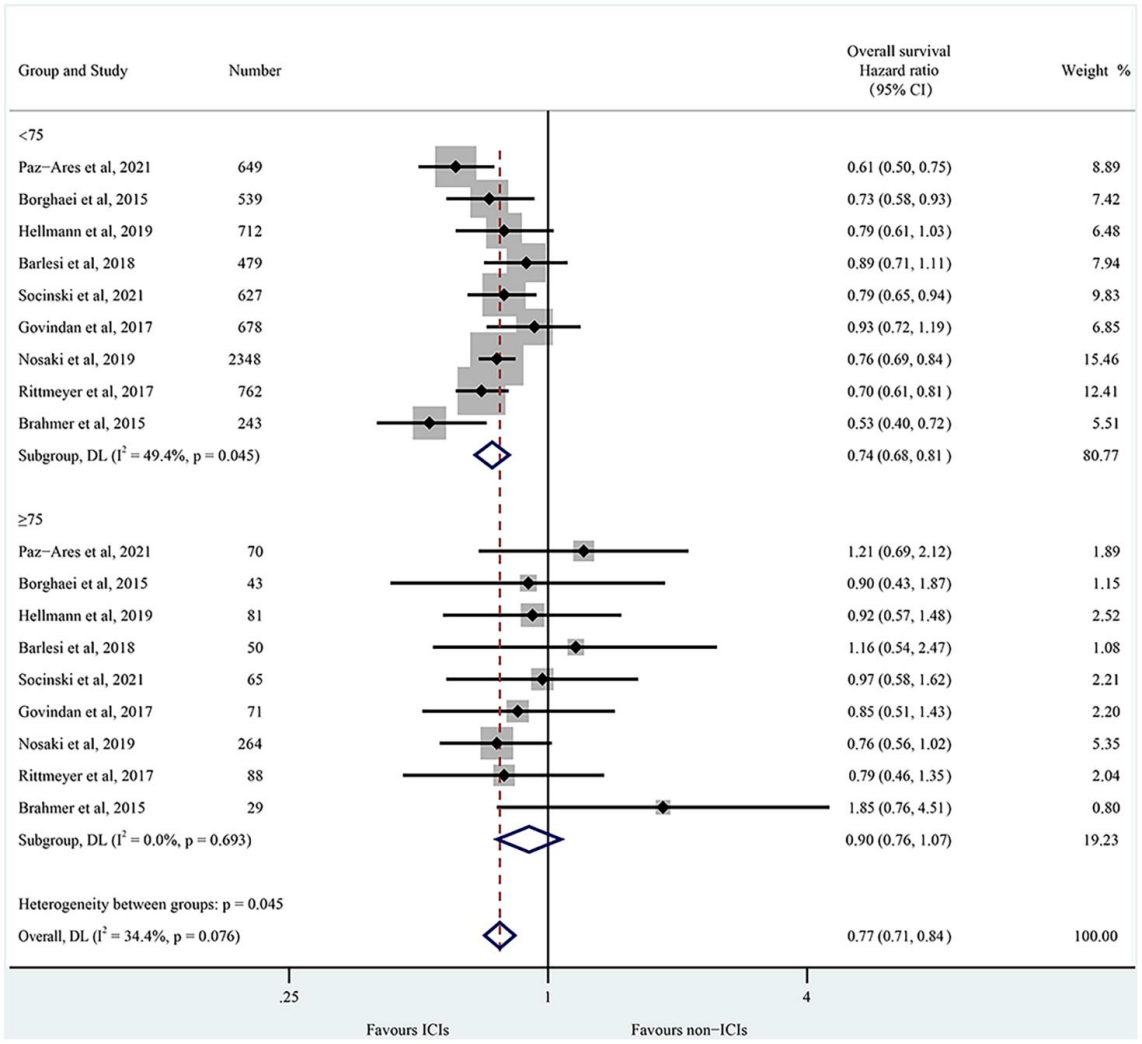
**Forest plots of overall survival of NSCLC patients < 75 years and ≥ 75 years by age.** CI, confidence interval; ICIs, immune checkpoint inhibitors.

The primary outcome of this study concerns OS from RCTs comparing ICIs to non-ICIs treatments. Supplementary Figure 3A depicts the HR of each trial and the pooled result based on the random-effects model. Overall, the estimated HR is 0.75 (95% CI: 0.69 to 0.81, P < 0.001), indicating that ICIs treatment resulted in a 25% reduction in the risk of death when compared to non-ICIs treatment. However, individual trials observed significant heterogeneity (I^2^= 47.3%, chi-squared P= 0.041). Patients were then segregated into two age groups with a cutoff age of 75 years. For non-elderly patients, the estimated HR for OS between ICIs and chemotherapy is 0.74 (95% CI: 0.68 to 0.81) (Figure 3). Significant heterogeneity was observed among individual trials in this cohort (I^2^= 49.4%, chi-squared P= 0.045). Conversely, the random-effects estimate of the HR for those aged 75 or older amounts to 0.90 (95% CI 0.76 to 1.07) (Figure 3), and there was no significant heterogeneity in this group (I^2^= 0.0%, chi-squared P= 0.693). We used independent samples T-tests to calculate OS for NSCLC patients in the non-elderly and elderly groups. Our results concluded that there was a significant difference in efficacy between non-elderly and elderly patients (P= 0.025). In addition, the relevant factors affecting the efficacy of ICIs were summarized and presented in Supplementary Table 5.

Funnel plots for the risk ratio of the 75-year-old age threshold and efficacy were shown in Supplementary Figure 4. The funnel plot analysis was symmetrical, and the p-value of the bias test is > 0.05, thereby identifying no evidence of publication bias in this study. When one trial was removed from the analyses for OS, the corresponding pooled HRs were not significantly altered, indicating that the presented results were relatively stable (Supplementary Figure 5). Subsequently, random-effects meta-regression analysis was performed, and a p-value of 0.089 was observed in terms of OS between the age groups.

### Secondary outcome: progression-free survival

Among studies included in this analysis, 11 had HR for PFS, including five based on age groups. The combined results based on the random-effects model estimated HR for PFS is 0.79 (95% CI: 0.70 to 0.89; P < 0.001) (Supplementary Figure 3B). The chi-squared test for heterogeneity was highly significant (I^2^= 78.1%, P < 0.001). The random-effects estimate of the HR of ICIs compared with chemotherapy in the non-elderly group is 0.82 (95% CI: 0.68 to 1.00; P= 0.045; Supplementary Figure 6). However, the HR estimate for elderly patients is 0.96 (95% CI: 0.70 to 1.32; P= 0.802; Supplementary Figure 6). For the non-elderly group, there is substantial heterogeneity (I^2^= 71.3%, chi-squared P= 0.008); but for the elderly group, there was no heterogeneity (I^2^= 5.4%, chi-squared P= 0.376).

### Safety analysis

**Figure 4:**
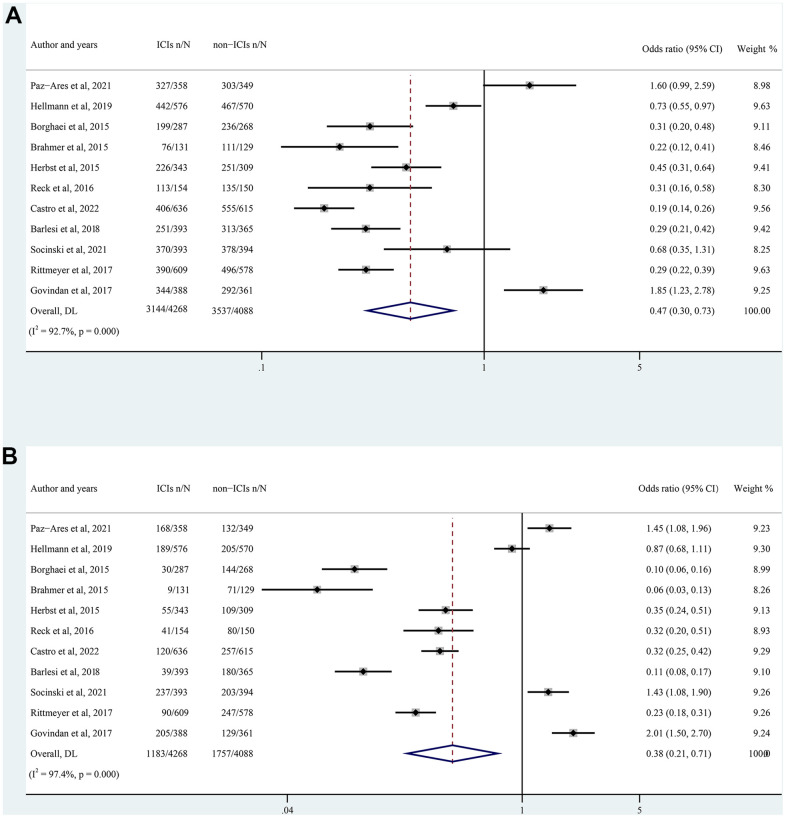
Forest plot of all-grade (**A**) and high-grade (**B**) TRAEs in NSCLC patients treated with ICIs versus non-ICIs. CI, confidence interval; TRAEs, treatment-related adverse events; ICIs, immune checkpoint inhibitors.

A total of 11 RCTs were included for safety analysis, including the number of patients with all-grade and high-grade TRAEs in the intervention group (ICIs group) and the control group (chemotherapy group). In terms of ICIs in NSCLC patients, there was a significantly lower incidence of all-grade TRAEs than non-ICIs (OR= 0.47; 95% CI: 0.30 to 0.73; p < 0.0001) (Figure 4A). Compared with non-ICIs, ICIs result in a lower incidence of high-grade TRAEs (OR= 0.38; 95% CI: 0.21 to 0.71; p < 0.0001) (Figure 4B). We next performed subgroup analysis, given the significant heterogeneity observed (I^2^= 92.7% and 97.4%, respectively).

### Subgroup analysis

Efficacy subgroup analysis was conducted in the elderly group based on ECOG PS, types of ICIs, and treatment lines. Firstly, we estimated the effect of ECOG PS on OS. The HR for OS in the PS= 0 subgroup was 0.73 (95% CI: 0.62 to 0.85), and in the PS= 1 subgroup was 0.81 (95% CI: 0.74 to 0.89). There was no heterogeneity in the two subgroups (chi-squared P= 0.135 and P= 0.317, respectively) (Supplementary Figure 7). Subsequently, we evaluated the effects of different types of ICIs. OS data were available from 8 trials for the anti-PD-1/L1 treatment, 1 for the anti-CTLA-4 treatment, and 2 for the combination treatment. The HR for OS in the anti-PD-1/L1 subgroup was 0.74 (95% CI: 0.67 to 0.82) in the non-elderly group and 0.87 (95% CI: 0.71 to 1.07) in the elderly group. In the anti-CTLA-4 subgroup, the HR for OS was 0.93 (95% CI: 0.72 to 1.02) in the non-elderly group and 0.85 (95% CI: 0.51 to 1.42) in the elderly group. In the combination subgroup, the HR for OS was 0.68 (95% CI: 0.53 to 0.88) in the non-elderly group and 1.03 (95% CI: 0.72 to 1.48) in the elderly group (Supplementary Table 4). In the end, elderly and non-elderly NSCLC patients were categorized into first-line and second-line subgroups, respectively, to explore the OS benefit from different treatment lines in NSCLC patients of different ages. For non-elderly NSCLC patients, the HR for OS was 0.74 (95% CI: 0.66 to 0.82) in the first-line subgroup and 0.75 (95% CI: 0.63 to 0.88) in the second-line subgroup (Supplementary Figure 8A), which did not show significant difference. For elderly NSCLC patients (aged ≥ 75 years), HRs were 0.88 (95% CI: 0.71 to 1.08) and 0.95 (95% CI: 0.71 to 1.27) for the first- and second-line subgroups, respectively (Supplementary Figure 8B), which were also not significantly different.

Safety subgroup analysis regarding all-grade TRAEs showed that compared to chemotherapy, anti-PD-1/L1 antibodies such as nivolumab, pembrolizumab, avelumab, and atezolizumab resulted in a lower incidence of TRAEs with ORs of 0.27 (95% CI: 0.19 to 0.39), 0.29 (95% CI: 0.16 to 0.53), 0.29 (95% CI: 0.21 to 0.42), and 0.42 (95% CI: 0.19 to 0.96), respectively. However, TRAEs were higher in the dual immunotherapy as well as ipilimumab, with ORs of 1.05 (95% CI: 0.49 to 2.28) and 1.85 (95% CI: 1.23 to 2.78), respectively (Supplementary Figure 9A). Data on high-grade TRAEs varied slightly, with nivolumab and avelumab exhibiting extremely low incidences of TRAEs, with ORs of 0.09 and 0.11, respectively. Anti-CTLA-4 antibodies (ipilimumab), however, had more than twice the incidence of TRAEs than chemotherapy (OR= 2.01) (Supplementary Figure 9B and Table 3).

**Table 3:** Subgroup analysis for different types of ICIs in terms of the incidence of all-grade and high-grade TRAEs in the ICIs group and chemotherapy group.

**Type of immunotherapy**	**Number of studies**	**OR (95% CI)**	
		**All-grade TRAEs**	**High-grade TRAEs**
Nivolumab	2	0.27 (0.19 to 0.39)	0.09 (0.05 to 0.14)
Pembrolizumab	3	0.29 (0.16 to 0.53)	0.33 (0.27 to 0.40)
Avelumab	1	0.29 (0.21 to 0.42)	0.11 (0.08 to 0.17)
Atezolizumab	2	0.42 (0.19 to 0.96)	0.58 (0.10 to 3.42)
Ipilimumab	1	1.85 (1.23 to 2.78)	2.01 (1.50 to 2.70)
Nivolumab plus Ipilimumab	2	1.05 (0.49 to 2.28)	1.12 (0.67 to 1.85)

## DISCUSSION

Considering that few high-level studies have pooled the efficacy of ICIs in NSCLC patients aged ≥ 75 years and those patients account for more than 1/3 of clinical practice. In this study, NSCLC patients were divided into two groups based on age: those aged < 75 years old and those aged ≥ 75 years old. Overall, both the retrospective study and meta-analysis showed statistically differences in OS between the two groups (P= 0.029 and 0.025, respectively), indicating that age may have a considerable effect on the efficacy of ICIs. In non-elderly NSCLC patients, ICIs provide a significant survival benefit (HR for OS: 0.74 and PFS: 0.82), but have reduced efficacy in elderly patients (HR for OS: 0.90, for PFS: 0.96). In addition, our retrospective study revealed that ECOG PS was negatively correlated with OS and PFS, patients with higher PS having lower OS and PFS, consistent with previous study [21]. On the other hand, this meta-analysis also showed PS of 0 or 1 barely had impact on OS in elderly NSCLC patients. We speculate that this may be due to RCTs' strict patient inclusion criteria, in which NSCLC patients with poor general conditions are usually excluded, thus, the meta-analysis did not show that ECOG was associated with poorer OS. Regarding safety analysis, ICIs resulted in a 53% lower incidence of all-grade TRAEs and 62% high-grade TRAEs than chemotherapy. In addition, subgroup analysis revealed that elderly patients tend to have better outcomes when classified according to the types of ICIs, especially those treated with PD-1/L1 inhibitors.

ICIs have demonstrated superior efficacy in NSCLC patients [22], but many studies have conflicting conclusions for elderly NSCLC patients. Several studies have shown that ICIs remain highly effective in elderly patients. Nishijima et al. [23] proposed that ICIs had significant OS for younger and older patients aged 65-70. In addition, a meta-analysis found that first-line ICIs treatment for patients aged ≥75 years with advanced tumors appears to be effective [24]. However, more studies indicated that ICIs were less effective in elderly patients. Age-stratified results from KEYNOTE-010 and KEYNOTE-042 studies showed significantly lower survival benefits in patients with advanced NSCLC aged ≥ 75 years than those aged < 75 years [25]. A multicenter retrospective cohort study explored OS trends in advanced NSCLC patients treated with ICIs at different ages [5]. The results showed that the population with the most significant survival benefit in the ICIs era was patients under 75 years old, and age was negatively correlated with OS. Even Takigawa et al. [9] pointed out that ICIs were not shown to be more effective than chemotherapy alone in NSCLC patients aged ≥ 75 years. Our findings partially support the notion that ICIs show reduced efficacy in elderly NSCLC patients.

The phenomenon of ICIs leading to reduced efficacy in elderly NSCLC patients actually makes sense. Elderly patients have a completely different immune microenvironment, including increased tumor mutation burden (TMB), increased expression of immune checkpoint genes, and low T-cell receptor diversity. Regarding drug mechanism of action, ICIs exert anti-tumor effects mainly by blocking specific immune checkpoints on T cells and primarily rely on the patient’s immune system [26]. The immune system of elderly patients exhibits decreased immunosurveillance and antigen presentation. The reduced efficacy of ICIs in elderly patients may be partially related to immunosenescence. Immunosenescence may not only be associated with an increased risk of cancer progression, but may also make patients less responsive to ICIs by upregulating immunosuppressive signaling [8, 27]. Immunosenescence is characterized by a decrease in the peripheral pool of naive T cells and T cell receptors and changes in the composition of the regulatory T cell population [28]. Key factors that stimulate T-cell activation, such as interferon-α (IFN-α) and interleukin-12 (IL-12), are secreted less in elderly patients, resulting in reduced dendritic cells (DCs) maturation [29]. CD8^+^ T cells are the main immune cells targeted by ICIs. As patients aging, both the initial T-lymphocyte population and the diversity of T-cell receptor (TCR) decrease, all of which interfere with the normal functioning of CD8^+^ T cells [30]. In addition, regulatory T cells (Tregs) with immunosuppressive effects increase with age, causing a further decline in anti-tumor immunity in elderly NSCLC patients [31]. Thus, elderly NSCLC patients may respond differently to ICIs than younger patients. Moreover, elderly patients have poorer ECOG PS (usually ≥ 2) due to comorbidities of multiple chronic diseases. NSCLC patients with impaired PS treated with ICIs were twice less likely to achieve a response compared to the general population [21]. Combined with impaired T cells and ECOG PS, the theoretical basis supports the reduced efficacy of ICIs in elderly NSCLC patients.

However, our study also had some limitations. First, the limited number of aged ≥ 75 years NSCLC patients included in the retrospective study and meta-analysis may have affected the generalizability of our findings. Additionally, the follow-up duration included in our study was relatively short, so we could not determine the long-term efficacy of ICIs in elderly NSCLC patients. Furthermore, the safety meta-analysis explored the incidence of TRAEs in the overall population with ICIs and chemotherapy, but not in elderly patients ≥ 75 years, due to insufficient data in the original literature. In fact, elderly patients are usually associated with poorer general conditions in the real world. Thus, ICIs treatment in this population requires comprehensive consideration.

In conclusion, our study demonstrates that ICIs are an effective treatment for non-elderly NSCLC patients. However, ICIs may have reduced efficacy in elderly NSCLC patients, especially those aged 75 years or older. Moreover, ICIs resulted in a substantially lower incidence of TRAEs than chemotherapy, indicating that ICIs are a safer treatment for NSCLC patients. For elderly NSCLC patients, sometimes it is not the tumor that leads to death but the toxic side-effects caused by the treatment. Therefore, we should consider the efficacy and evaluate it in the context of TRAEs. NSCLC patients with poorer general conditions (higher ECOG PS) should be excluded from ICIs treatment. When choosing ICIs for elderly patients, the type of ICIs should be considered, with priority given to PD-1/L1 inhibitors such as pembrolizumab and nivolumab. Real-world and prospective studies evaluating the efficacy of ICIs in elderly NSCLC patients are needed to formulate the best treatment strategy and break through the status quo in the treatment of elderly NSCLC.

## MATERIALS AND METHODS

### Retrospective efficacy study

### Data source

The cBioPortal for Cancer Genomics database (http://www.cbioportal.org/) contains raw data on more than 20,000 tumor samples from multiple cancer studies. The cBio website includes not only molecular profiles of cancer genomics projects, but also information on patients' clinical data. Among them are 29 datasets on NSCLC studies containing a total of 13,690 NSCLC patients.

### Search strategy and data extraction

We performed a retrospective analysis using the public dataset available on the cBio website. The inclusion criteria were as follows: (i) NSCLC patients, (ii) treatment with ICIs, and (iii) survival data such as OS or PFS were available. Patients with NSCLC who had surgical resection were excluded from this study. Data pertaining to case ID, demographics (age, sex, ECOG PS), tumor histology type, types of ICIs, and survival data (OS, PFS) were analyzed.

### Data analysis

Descriptive statistics were used to describe demographic and clinical characteristics. Univariate analysis was performed using the Kaplan-Meier method to compare OS and PFS between patients' clinical characteristics (such as age, sex, ECOG PS, types of ICIs, and histology type). P < 0.05 was considered statistically significant. Parameters with P < 0.05 based on the univariate analysis were further included in the multivariate Cox regression analysis. All statistical analyses were performed using SPSS 26.0 software (IBM, Armonk, NY, USA).

### Meta-analysis

This meta-analysis (PROSPERO registration No. CRD42023394032) compared OS and PFS in elderly and non-elderly patients to investigate whether ICIs are still effective for elderly patients. The incidence of TRAEs due to ICIs and chemotherapy was calculated to indirectly reflect which treatment has lower toxicity in elderly NSCLC patients (who are usually in poor general condition).

### Search strategy and selection criteria

This meta-analysis followed the Preferred Reporting Items for Systematic Reviews and Meta-Analysis (PRISMA) [32]. A checklist of PRISMA items is presented in Supplementary Table 1. We conducted a comprehensive literature search of PubMed, Embase, ClinicalTrials.gov, and Cochrane Library databases to identify the relevant articles. The search spanned the dates of each database's inception through 2 July 2023. Search terms included the following keywords: “PD-1”, “programmed cell death protein 1”, “PD-L1”, “programmed cell death ligand 1”, “pembrolizumab”, “cemiplimab”, “nivolumab”, “avelumab”, “atezolizumab”, “durvalumab”, “CTLA-4”, “cytotoxic T-lymphocyte-associated protein 4”, “ipilimumab”, “tremelimumab”, “NSCLC”, “non-small cell lung cancer”, and “immune checkpoint inhibitors”, limiting to RCTs. The American Society of Clinical Oncology (ASCO) and European Society for Medical Oncology (ESMO) meeting database was also searched for additional studies. The detailed search strategies were shown in Supplementary Doc 1 (Supplementary Material).

The inclusion criteria were as follows: (i) RCTs in patients with NSCLC, (ii) Studies comparing the efficacy of ICIs to non-ICIs, (iii) Subgroup analysis of OS using a HR based on the age (≥ 75 years versus < 75 years). Two reviewers (YJX and SYX) independently screened the titles and abstracts to identify the potential articles, and then assessed the eligibility of the full texts of these relevant articles.

### Data extraction

Data extracted from eligible studies included characteristics of both the study (first author, publication year, study name and National Clinical Trial (NCT) number, trial phase, treatment arms) and the study population (total number of randomized patients, median age, number of patients aged < 75 years and ≥ 75 years, median follow-up duration). We collected data on HR with a 95% confidence interval (CI) for OS and PFS based on age subgroups (< 75 years vs. ≥ 75 years). Studies without HR by age group or excluded elderly patients were not included. Then, we extracted the number of reported events between all-grade and high-grade TRAEs from the experimental and control groups in each study to perform a safety analysis. In this study, grade ≥ 3 was considered as high-grade TRAEs. Disagreement in the literature search and data extraction was resolved by discussion between all authors of this meta-analysis.

### Quality of evidence

Search strategies were finally integrated with the Cochrane highly sensitive search strategy to identify RCTs. The methodological quality of the studies was assessed using the five-point Jadad scale, which mainly evaluated three aspects (randomization, blinding, withdrawals and dropouts) of all the studies [33]. A score of 0-2 was considered low quality, while a score of 3-5 was regarded as high quality.

### Data synthesis and analysis

Our analysis included two groups: non-elderly patients (aged < 75 years) and elderly patients (aged ≥ 75 years). A meta-analysis was conducted to quantify the effect of ICIs on OS and PFS using the HR with the corresponding 95% CI. All-grade and high-grade TRAEs were synthesized via meta-analysis using the Mantel–Haenszel model. We calculated the odds ratio (OR) and 95% CI between the experimental and control groups based on the number of reported events and sample size.

To account for the potential heterogeneity among the included trials, all the pooled HR of this meta-analysis was calculated using random-effects models. We combined the HR estimates for patients aged < 65 years and 65-75 years, and the combined estimate was used for the subsequent meta-analysis. The forest plots were utilized to summarize and visualize the HR and OR with 95% CI for each study. The DerSimoniane-Laird method was employed to examine the trials' heterogeneity and quantified by the I^2^ index and Chi-squared p-values. The studies were deemed substantially heterogeneous when P < 0.1 and I^2^ > 50%. Differences in the HRs between the non-elderly and elderly groups were assessed using an independent sample t-test, with a two-sided p-value of < 0.05 defined as significant.

To analyze the age effect in ICIs, subgroup analyses were conducted using the ECOG PS and types of ICIs (PD-1, PD-L1, CTLA-4, and a combination of two). A safety subgroup analysis was performed to assess which types of ICIs resulted in a lower incidence of TRAEs. Funnel plots were utilized to evaluate the presence of publication bias. A sensitivity analysis was performed to verify the stability of the summary results. Meta-regression was also conducted to investigate the effect of age differences on ICIs efficacy after adjusting for other factors. All the statistical analyses were performed using Stata version 17.0 (Stata Corp, College Station, TX, USA).

### Availability of data and materials

The datasets generated during and/or analyzed during the current study are available from the corresponding author on reasonable request.

## Supplementary Material

Supplementary Doc 1

Supplementary Figures

Supplementary Table 1

Supplementary Tables 2-5

Supplementary Appendix 1
