# Supplementary material for: The efficacy of immune checkpoint inhibitors is limited in elderly NSCLC: a retrospective efficacy study and meta-analysis
Source: Aging (Albany NY). 2023 Dec 19;15(24):15025–49. doi: 10.18632/aging.205328 (PMC10781456; doi:10.18632/aging.205328)
Supplement: Supplementary Doc 1 [file aging-15-205328-s001.docx]

**Supplementary Doc 1. Detailed search strategies for each database.**

PubMed=548

#1 (Lung Neoplasms [mh]) OR (Lung Neoplasms) OR (Neoplasms, Lung) OR (Lung Neoplasm) OR (Neoplasm, Lung) OR (Neoplasms, Pulmonary) OR (Neoplasm, Pulmonary) OR (Pulmonary Neoplasm) OR (Pulmonary Neoplasms) OR (Lung Cancer) OR (Cancer, Lung) OR (Cancers, Lung) OR (Lung Cancers) OR (Pulmonary Cancer) OR (Cancer, Pulmonary) OR (Cancers, Pulmonary) OR (Pulmonary Cancers) OR (Cancer of the Lung) OR (Cancer of Lung) OR (Carcinoma, Non-Small-Cell [mh]) OR (Carcinoma, Non-Small-Cell) OR (Carcinoma, Non Small Cell Lung) OR (Carcinomas, Non-Small-Cell Lung) OR (Lung Carcinoma, Non-Small-Cell) OR (Lung Carcinomas, Non-Small Cell) OR (Non-Small-Cell Lung Carcinomas) OR (Non-Small-Cell Lung Carcinoma) OR (Non Small Cell Lung Carcinoma) OR (Carcinoma, Non-Small Cell Lung) OR (Non-Small Cell Lung Cancer)

#2 (Immune Checkpoint Inhibitors [mh]) OR (Immune Checkpoint Inhibitor) OR (Programmed Cell Death 1 Receptor [mh]) OR (PD-1 Protein) OR (PD-1 Receptor) OR (Programmed Cell Death Protein 1) OR (Nivolumab [mh]) OR (Opdivo) OR (ONO-4538) OR (MDX-1106) OR (BMS-936558) OR (Pembrolizumab) OR (SCH 900475) OR (Lambrolizumab) OR (MK-3475) OR (Keytruda) OR (B7-H1 Antigen [mh]) OR (Programmed Death Ligand 1) OR (CD274 Antigens) OR (Programmed Cell Death 1 Ligand 1) OR (Avelumab) OR (Bavencio) OR (MSB-0010718C) OR (Atezolizumab) OR (MPDL-3280A) OR (Tecentriq) OR (RG-7446) OR (Durvalumab) OR (MEDI-4736) OR (Imfinzi) OR (CTLA-4 Antigen [mh]) OR (Cytotoxic T-Lymphocyte-Associated Antigen 4) OR (CD152 Antigens) OR (CTLA-4 Protein) OR (Ipilimumab [mh]) OR (Yervoy) OR (MDX-010) OR (MDX-CTLA-4) OR (Tremelimumab) OR (Ticilimumab) OR (CP-675)

#3 (Randomized Controlled Trial) OR (Randomized controlled trial) OR (RCT)

#4 ("0001/01/01"[Date - Publication]: "2023/01/02"[Date - Publication])

#5 #1 AND #2 AND #3 AND #4

EMBASE=6713

#1 'lung tumor'/exp OR 'non small cell lung cancer'/exp OR ((lung NEXT/1 carcinom*):ab,ti) OR ((lung NEXT/1 neoplasm*):ab,ti) OR 'lung cancer':ab,ti OR nsclc:ab,ti OR 'non small cell lung':ab,ti OR ((lung NEXT/1 tumour*):ab,ti) OR 'nonsmall cell lung':ab,ti OR 'non-small cell lung':ab,ti OR 'lung carcinoma'/exp OR 'lung cancer'/exp

#2 'programmed death 1 receptor'/exp OR 'programmed death 1 ligand 2'/exp OR 'programmed death 1 ligand 1'/exp OR 'anti programmed death':ab,ti OR 'programmed death ligand':ab,ti OR 'programmed death 2':ab,ti OR 'programmed death 1':ab,ti OR 'target* pd-l1':ab,ti OR 'target* pd-l2':ab,ti OR 'target* pd-1':ab,ti OR 'pd-l1 block*':ab,ti OR 'pd-l2 block*':ti,ab OR 'pd-1 block*':ab,ti OR 'pd-l1 inhibitor*':ab,ti OR 'pd-l2 inhibitor*':ab,ti OR 'pd-1 inhibitor*':ab,ti OR 'anti pd-l1':ab,ti OR 'anti pd-l2':ab,ti OR 'anti pd-1':ab,ti OR 'immunotherap*':ab,ti OR 'immunotherapy'/exp OR 'durvalumab':ab,ti OR 'avelumab':ab,ti OR 'atezolizumab':ab,ti OR 'pembrolizumab':ab,ti OR 'nivolumab':ab,ti OR 'immune checkpoint inhibitor*':ab,ti OR 'ono-4538':ti,ab OR 'bms-936558':ti,ab OR 'ipilimumab':ti,ab OR 'tremelimumab':ti,ab OR 'ticilimumab':ti,ab OR 'pembrolizumab':ti,ab OR 'lambrolizumab':ti,ab OR 'cytotoxic t lymphocyte antigen 4'/exp OR ctla4:ti,ab OR keytruda:ti,ab OR mdx1106:ti,ab OR opdivo:ti,ab OR 'mk 3475':ti,ab OR mpdl3280a:ti,ab OR yervoy:ti,ab OR 'mdx 010':ti,ab OR 'mdx 101':ti,ab OR 'cp 675,206':ti,ab OR 'chemotherap*':ab,ti OR 'chemotherapy'/exp OR 'antineoplastic agent'/exp

#3 'crossover procedure'/exp OR 'double-blind procedure'/exp OR 'randomized controlled trial'/exp OR 'single-blind procedure'/exp OR random* OR factorial* OR crossover* OR cross NEXT/1 over* OR placebo* OR doubl* NEAR/1 blind* OR singl* NEAR/1 blind* OR assign* OR allocat* OR volunteer*

#4 #1 AND #2 AND #3

ClinicalTrials.gov=1050

#1 non-small cell lung cancer

#2 nsclc

#3 immunotherapy

#4 PD-1

#5 PD-L1

#6 CTLA-4

CENTRAL search strategy=438

#1, MeSH descriptor: [Carcinoma, Non-Small-Cell Lung] explode all trees

#2, nsclc

#3, lung cancer*

#4, lung carcinom*

#5, lung neoplasm*

#6, lung tumor*

#7, lung tumour*

#8, non small cell*

#9, (#3 or #4 or #5 or #6 or #7) and (#8)

#10, #1 or #2 or #9 #11, MeSH descriptor: [Programmed Cell Death 1 Receptor] explode all trees

#12, Programmed Cell Death 1

#13, PD-1 Receptor

#14, CD279 Antigen*

#15, PD1 Receptor

#16, MeSH descriptor: [Programmed Cell Death 1 Ligand 2 Protein] explode all trees

#17, CD 273

#18, PD L2 Ligand

#19, B7 DC Ligand

#20, B7 DC Antigen*

#21, programmed death

#22, pd l1

#23, pd l2

#24, pd 2

#25, MeSH descriptor: [Immunotherapy] explode all trees

#26, Immunotherap*

#27, durvalumab

#28, MEDI4736

#29, MEDI-4736

#30, Imfinzi

#31, avelumab

#32, MSB0010718C

#33, atezolizumab

#34, MPDL3280A

#35, Tecentriq

#36, RG7446

#37, RG 7446

#38, pembrolizumab

#39, lambrolizumab

#40, Keytruda

#41, MK3475

#42, MK 3475

#43, nivolumab

#44, MDX-1106

#45, ONO-4538

#46, BMS-936558

#47, Opdivo

#48, immune checkpoint inhibitor*

#49, MeSH descriptor: [Ipilimumab] explode all trees

#50, Ipilimumab

#51, Yervoy

#52, MDX010

#53, MDX 010

#54, tremelimumab

#55, ticilimumab

#56, CP 675*

#57, CP675*

#58, #11 or #12 or #13 or #14 or #15 or #16 or #17 or #18 or #19 or #20 or #21 or #22 or #23 or #24 or #25 or #26 or #27 or #28 or #29 or #30 or #31 or #32 or #33 or #34 or #35 or #36 or #37 or #38 or #39 or #40 or #41 or #42 or #43 or #44 or #45 or #46 or #47 or #48 or #49 or #50 or #51 or #52 or #53 or #54 or #55 or #56 or #57 #59, #10 and #5
