# Supplementary material for: The efficacy of immune checkpoint inhibitors is limited in elderly NSCLC: a retrospective efficacy study and meta-analysis
Source: Aging (Albany NY). 2023 Dec 19;15(24):15025–49. doi: 10.18632/aging.205328 (PMC10781456; doi:10.18632/aging.205328)
Supplement: Supplementary Figures [file aging-15-205328-s002.pdf]

SUPPLEMENTARY FIGURES

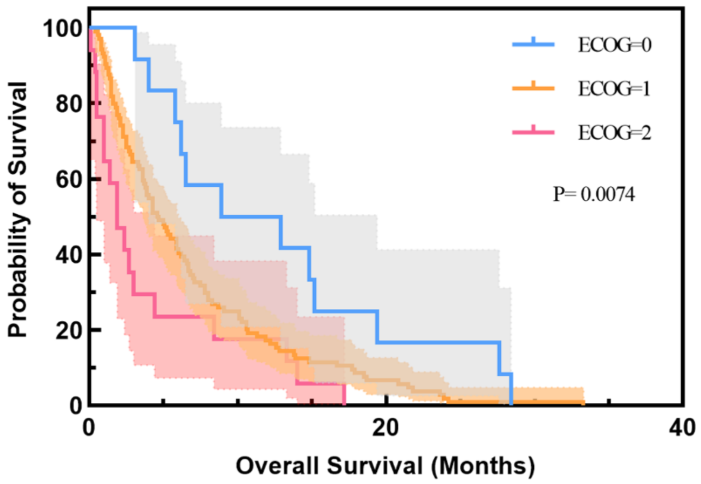

Supplementary Figure 1. Overall survival of NSCLC patients treated with ICIs based on ECOG PS in the cBio database.

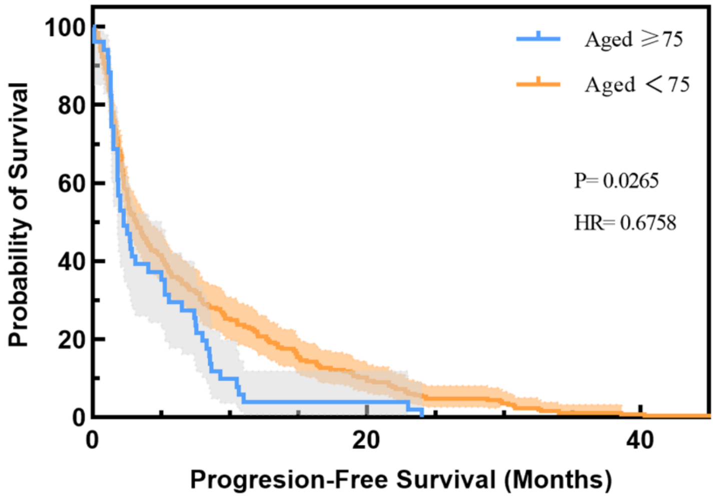

Supplementary Figure 2. Progression-free survival of NSCLC patients treated with ICIs based on age in the cBio database.

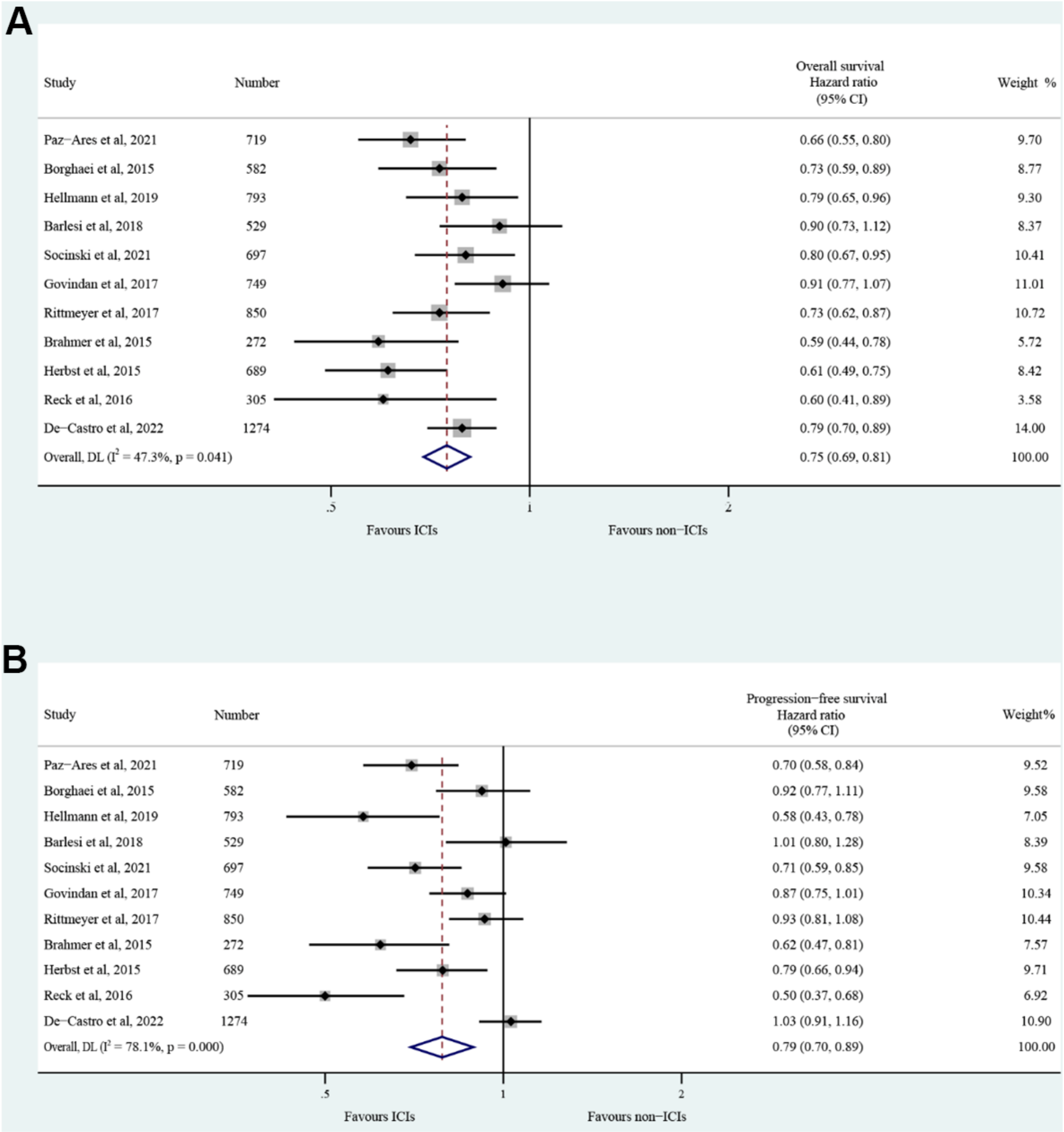

Supplemental Figure 3. Forest plot of the hazard ratio for overall survival (**A**) and progression-free survival (**B**). CI, confidence interval; ICIs, immune checkpoint inhibitors.

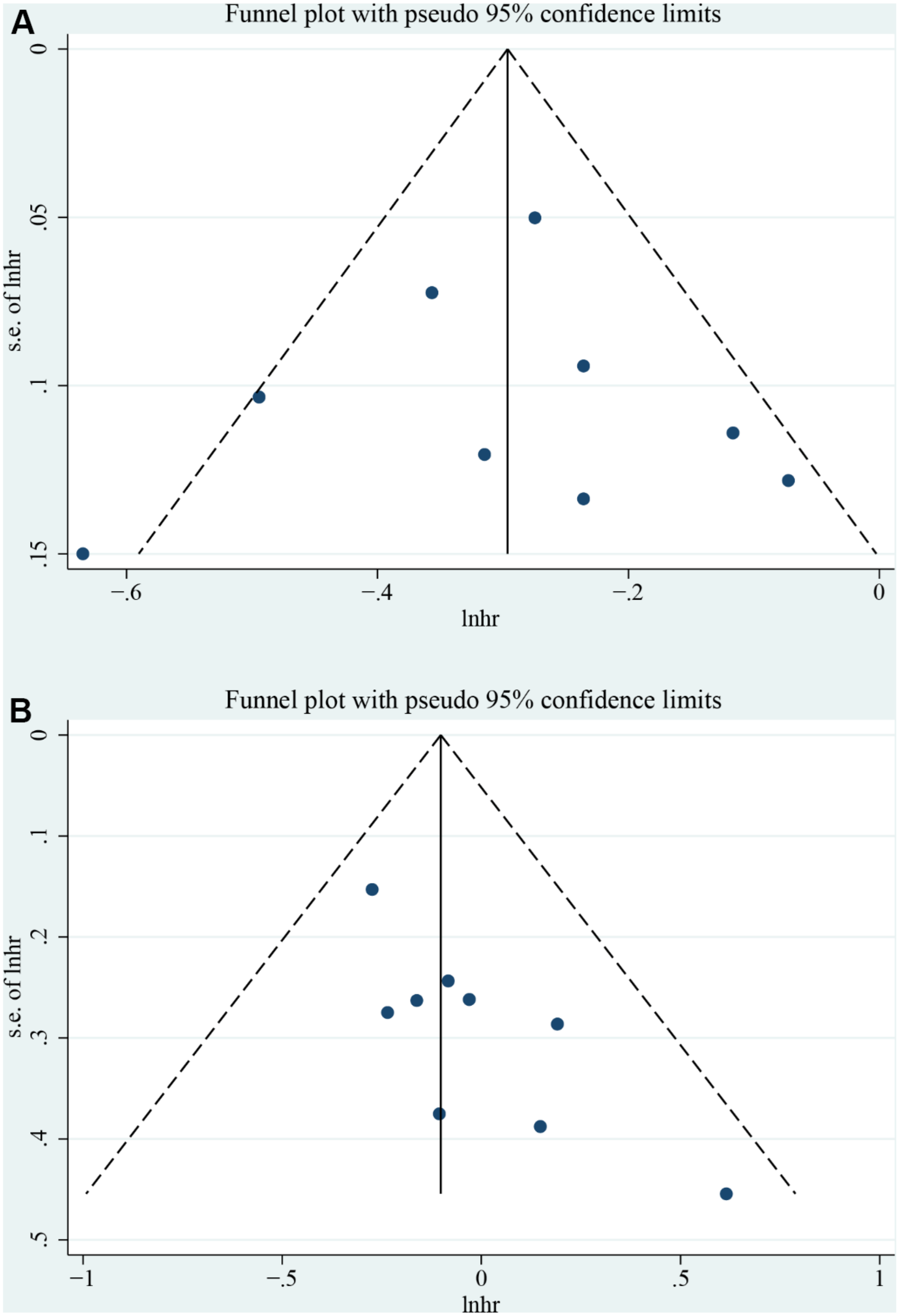

Supplementary Figure 4. Funnel plots of efficacy by age < 75 years (**A**) and ≥ 75 years (**B**).

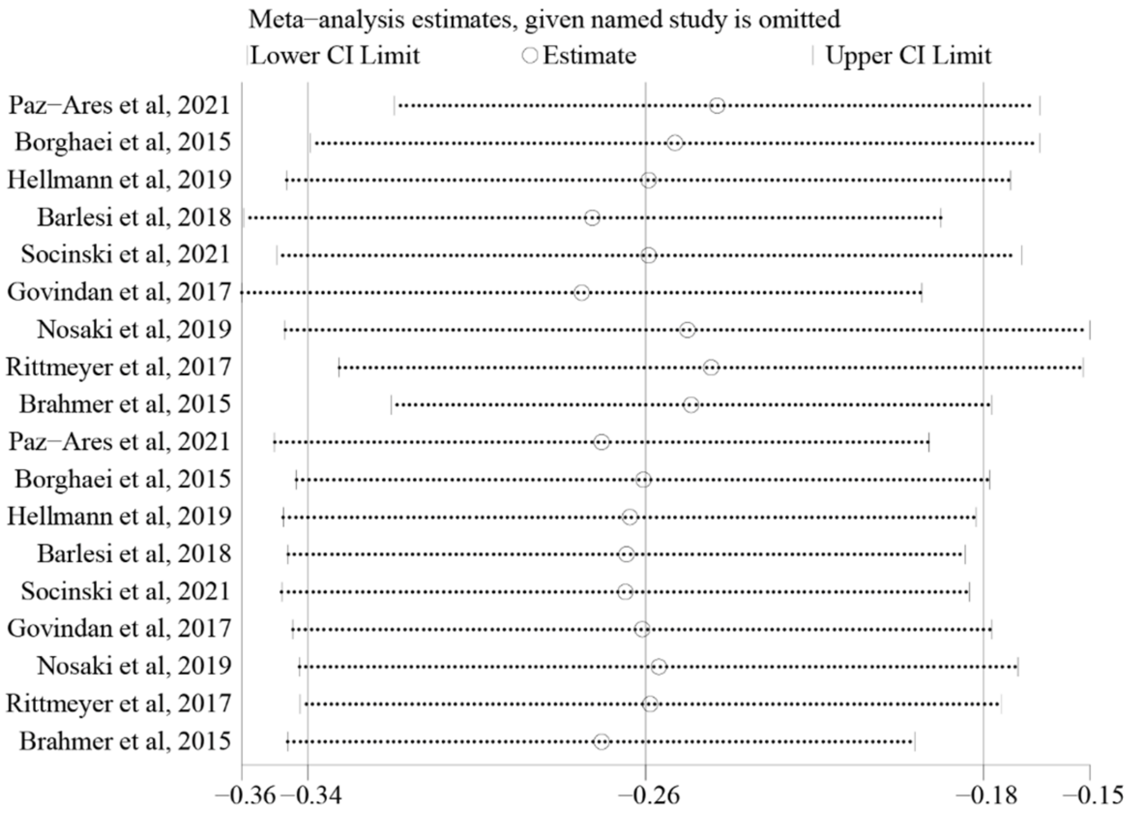

Supplementary Figure 5. Sensitivity analysis for OS at 75 years of age threshold. CI, confidence interval.

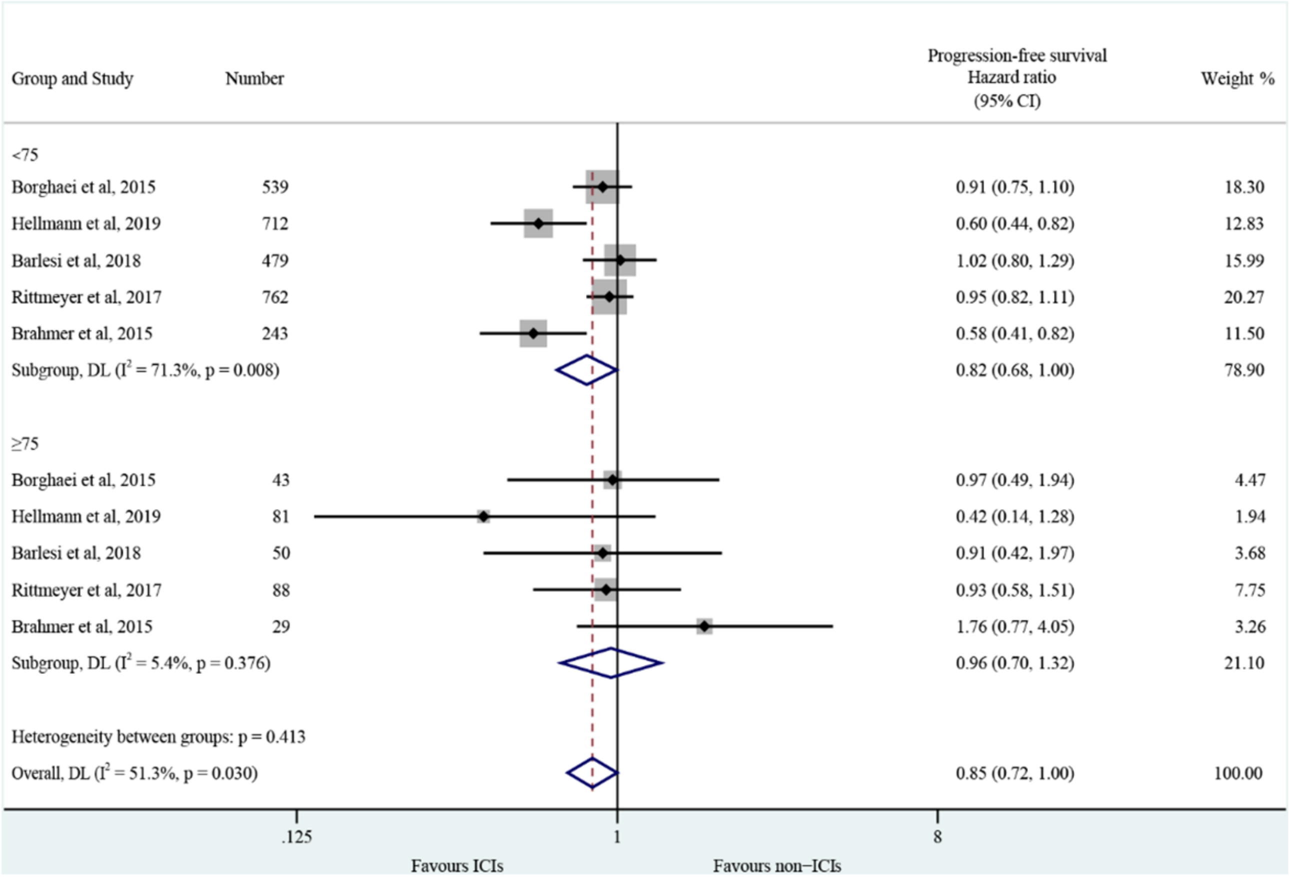

Supplementary Figure 6. Forest plots of progression-free survival by age < 75 years and ≥ 75 years. CI, confidence interval; ICIs, immune checkpoint inhibitors.

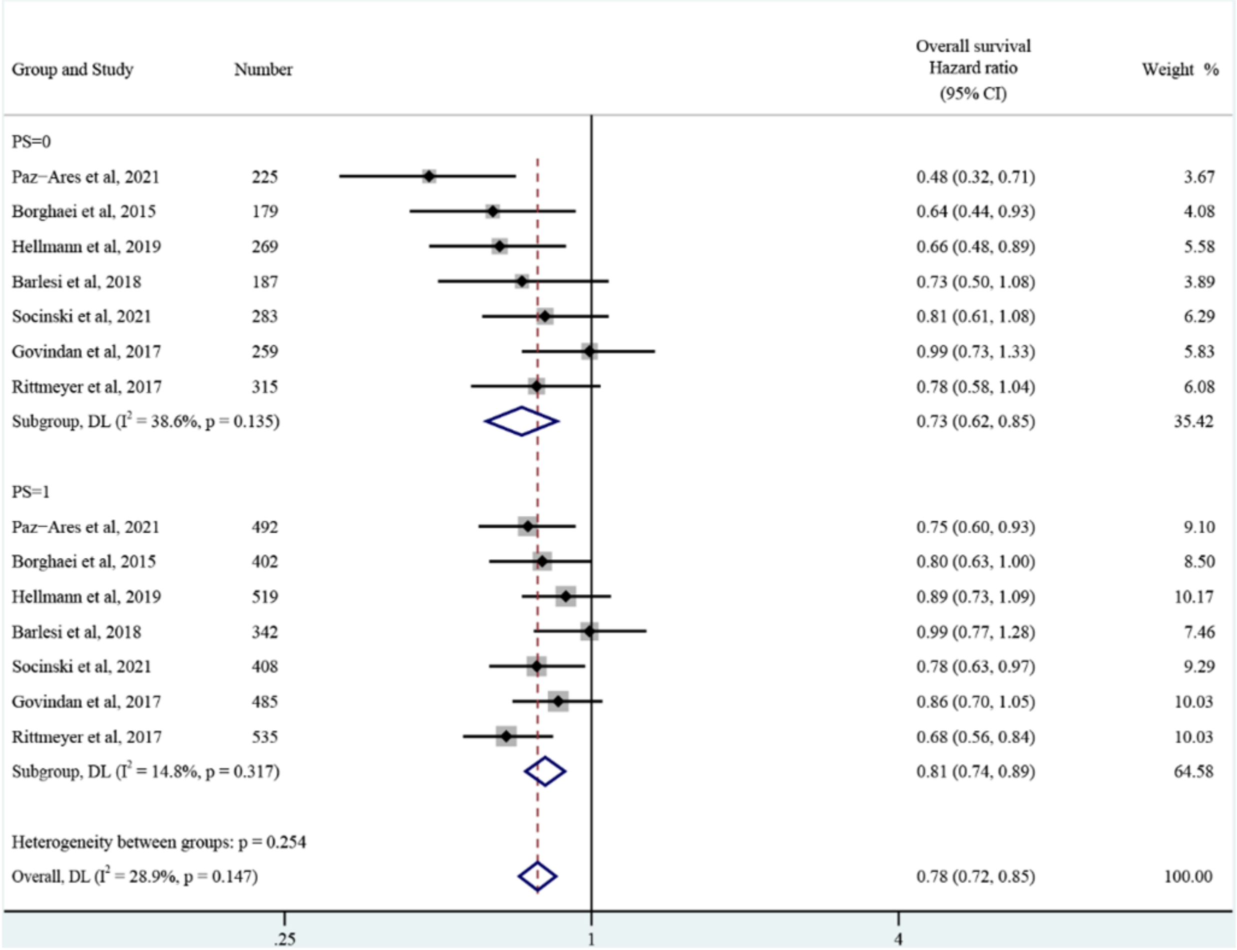

Supplementary Figure 7. Forest plots of overall survival by PS= 0 and PS= 1. CI, confidence interval; PS, performance status.

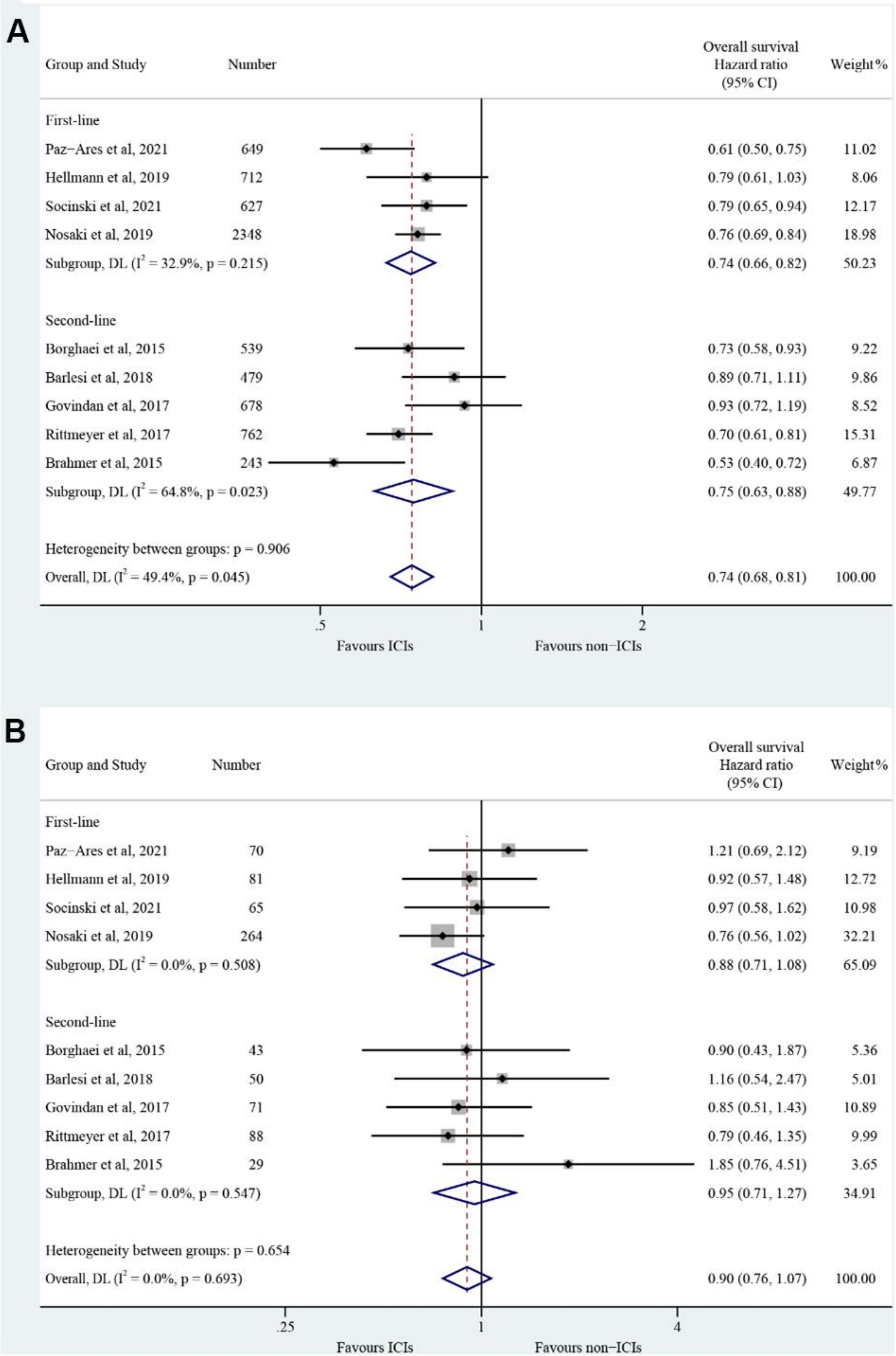

Supplementary Figure 8. Forest plots of aged <75 years old (**A**) and ≥75 years old (**B**) of overall survival in subgroups by treatment lines. CI, confidence interval; ICIs, immune checkpoint inhibitors.

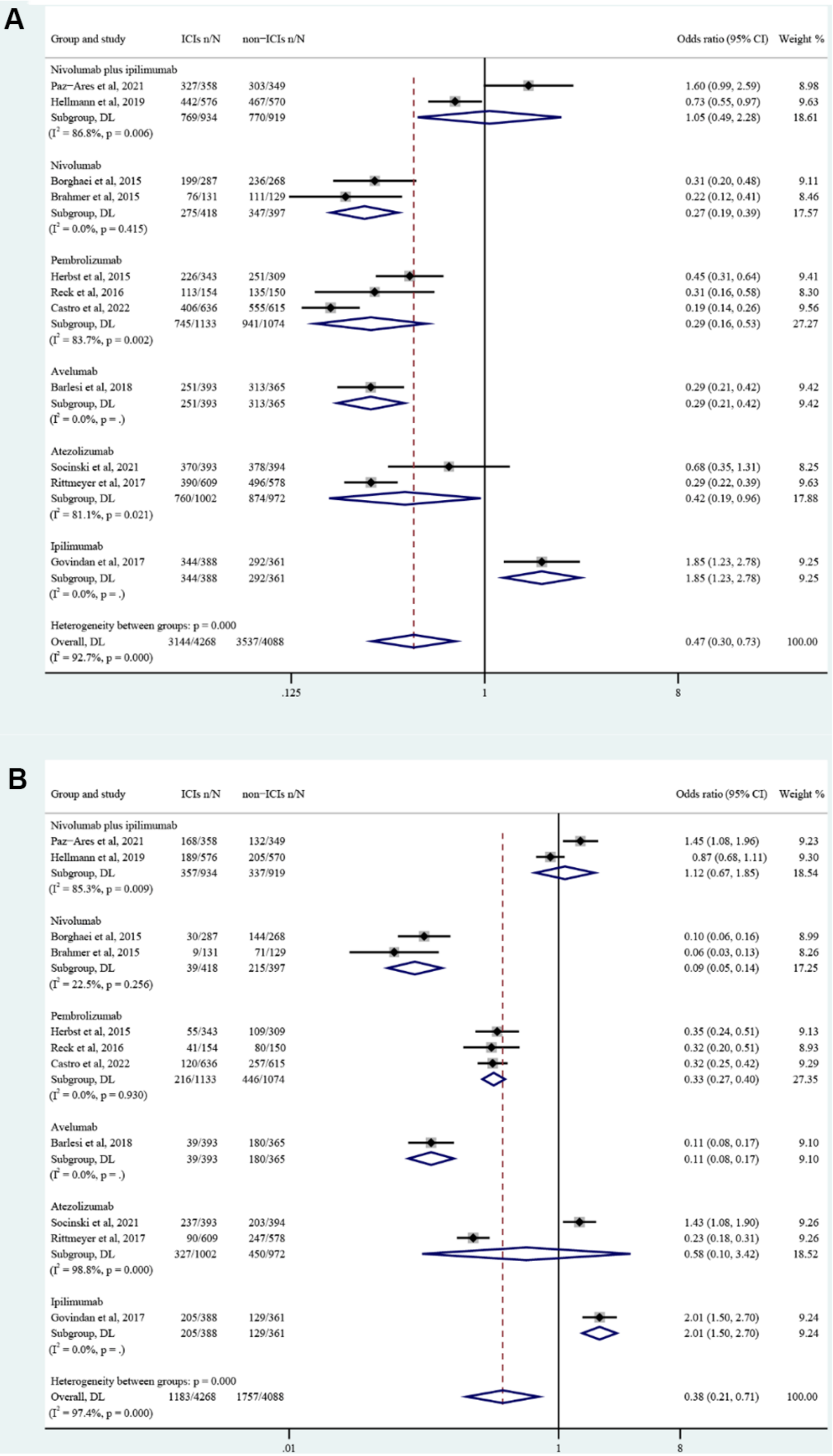

Supplementary Figure 9. Forest plots of all-grade (**A**) and high-grade (**B**) TRAEs in subgroups by type of ICIs. CI, confidence interval; ICIs, immune checkpoint inhibitors.
