# Supplementary material for: The efficacy of immune checkpoint inhibitors is limited in elderly NSCLC: a retrospective efficacy study and meta-analysis
Source: Aging (Albany NY). 2023 Dec 19;15(24):15025–49. doi: 10.18632/aging.205328 (PMC10781456; doi:10.18632/aging.205328)
Supplement: Supplementary Tables 2-5 [file aging-15-205328-s004.pdf]

Supplementary Table 2. Characteristics and univariate analysis of mPFS for NSCLC patients treated with ICIs from the cBio database.

Clinical characteristic	Patient and percentage (%)	mPFS (months)	p-Value
Total case	296 (100%)	2.9	-
Age			
Aged < 75 years old	245 (82.8%)	3.2	0.027*
Aged ≥ 75 years old	51 (17.2%)	2.3	
Sex			
Male	135 (45.6%)	2.9	0.940
Female	161 (54.4%)	3.0	
Tumor Histology			
Adenocarcinoma	247 (83.4%)	3.0	0.662
Squamous	49 (16.6%)	2.9	
ECOG PS			
ECOG 0	51 (17.2%)	6.8	< 0.001***
ECOG 1	220 (74.3%)	2.7	
ECOG 2	24 (8.1%)	1.6	
ECOG 3	1 (0.4%)	0.6	
Types of ICIs			
Anti-PD-1	174 (58.8%)	2.7	0.020*
Anti-PD-L1	43 (14.5%)	2.1	
Anti-CTLA-4	77 (26.0%)	4.8	
Anti-PD-1/L1 + Anti-CTLA-4	2 (0.7%)	0.6	

mPFS, median progression-free survival; *$P < 0.05$, ***$P < 0.001$.

Supplementary Table 3. Summary of Jadad score by randomization, blinding, and account.

Study (Year)	Randomization	Blinding	Account	Jadad score
Paz-Ares et al., 2021	2	0	1	3
Borghaei et al., 2015	2	0	1	3
Hellmann et al., 2019	2	0	1	3
Barlesi et al., 2018	2	0	1	3
Socinski et al., 2021	2	0	1	3
Govindan et al., 2017	2	0	1	3
Rittmeyer et al., 2017	2	0	1	3
Brahmer et al., 2015	2	0	1	3
Herbst et al., 2015	2	2	1	5
Reck et al., 2016	2	2	1	5
De-Castro et al., 2022	2	0	1	3

Notes: A score of 0–2 is considered low, and a score of 3–5 is defined as high quality.

Supplementary Table 4. Subgroup analysis for different types of ICIs in terms of OS between the non-elderly group (< 75 years) and elderly group (≥ 75 years).

Types of ICIs	Number of studies	HR (95% CI)	
		Age < 75 years	Age ≥ 75 years
Anti-PD-1/L1 antibody	8	0.74 (0.67 to 0.82)	0.87 (0.71 to 1.07)
Anti-CTLA-4 antibody	1	0.93 (0.72 to 1.20)	0.85 (0.51 to 1.42)
Anti-PD-1/L1 + Anti-CTLA-4	2	0.68 (0.53 to 0.88)	1.03 (0.72 to 1.48)

ICIs, immune checkpoint inhibitors; HR, hazard ratio; CI, confidence interval.

Supplementary Table 5. Summary of factors affecting the efficacy of ICIs.

Studies	Study number	Treatment lines	PD-L1 expression and its corresponding mOS				HR (95% CI)
			ICIs group	mOS	Chemotherapy group	mOS	
Paz-Ares, 2021	CheckMate 9LA (NCT03215706)	First-line	<1%: 135	<1%: 16.8 (13.7-NR)	<1%:129	<1%: 9.8 (7.7-13.7)	0.62 (0.45-0.85)
			≥1%: 203	≥1%:15.8 (13.8-NR)	≥1%:204	≥1%:10.9 (9.5-13.2)	0.64 (0.50-0.82)
			1-49%: 127	1-49%: 15.4 (12.6-NR)	1-49%:106	1-49%: 10.4 (8.7-12.4)	0.61 (0.44-0.84)
			≥50%: 76	≥50%:18.0 (13.1-NR)	≥50%:98	≥50%:12.6 (9.4-16.9)	0.66 (0.44-0.99)
Hellmann, 2019	CheckMate 227 (NCT02477826)	First-line	<1%: 187	<1%: 17.2 (12.8-22.0)	<1%: 186	<1%: 12.2 (9.2-14.3)	0.62 (0.49-0.79)
			≥1%: 396	≥1%: 17.1 (15.0-20.1)	≥1%: 397	≥1%: 14.9 (12.7-16.7)	0.79 (0.65-0.96)
			≥50%: 205	≥50%:21.2 (15.5-38.2)	≥50%: 192	≥50%:21.2 (15.5-38.2)	0.70 (0.55-0.90)
Barlesi, 2018	JAVELIN Lung 200 (NCT02395172)	Second-line	≥1%: 264	≥1%:11.4 (9.4-13.9)	≥1%:265	≥1%:10.3 (8.5-13.0)	0.90 (0.73-1.12)
			≥50%: 168	≥50%:13.6 (10.1-18.5)	≥50%:147	≥50%:9.2 (5.8-12.4)	0.67 (0.51-0.89)
			≥80%: 120	≥80%:17.1 (10.6-25.0)	≥80%:106	≥80%:9.3 (5.8-13.1)	0.59 (0.42-0.83)
Herbst, 2015	KEYNOTE 010 (NCT01905657)	Second-line	1-49%: 195	1-49%: 12.7 (10.0-17.3)	1-49%: 152	1-49%: 8.5 (7.5-9.8)	0.76 (0.60-0.96)
			≥50%: 151	≥50%:17.3 (11.8-NR)	≥50%: 191	≥50%:8.2 (6.4-10.7)	0.53 (0.40-0.70)
De-Castro, 2022	KEYNOTE 042 (NCT02220894)	First-line	≥1%: 637	≥1%:16.4 (14.0-19.6)	≥1%: 637	≥1%:12.1 (11.3-13.3)	0.79 (0.70-0.89)
			≥20%: 413	≥20%:18.0 (15.5-21.5)	≥20%: 405	≥20%:13.0 (11.6-15.3)	0.75 (0.64-0.87)
			≥50%: 299	≥50%:20.0 (15.9-24.2)	≥50%: 300	≥50%:12.2 (10.4-14.6)	0.68 (0.57-0.81)

mOS, median overall survival; ICIs, immune checkpoint inhibitors; HR, hazard ratio; CI, confidence interval; NR, not reached.
